# Monophasic Variant of *Salmonella* Typhimurium Infection Affects the Serum Metabolome in Swine

**DOI:** 10.3390/microorganisms11102565

**Published:** 2023-10-15

**Authors:** Guillaume Larivière-Gauthier, Annaëlle Kerouanton, Sophie Mompelat, Stéphanie Bougeard, Martine Denis, Philippe Fravalo

**Affiliations:** 1 USC Metabiot, Cnam, 22440 Ploufragan, France; gullaume.lariviere-gauthier@lecnam.net; 2 USC Metabiot, Anses, Ploufragan-Plouzané-Niort Laboratory, Hygiene and Quality of Poultry and Pig Products Unit, 22440 Ploufragan, France; annaelle.kerouanton@anses.fr; 3 Anses, Fougères Laboratory, Analysis of Residues and Contaminants Unit, 35133 Fougères, France; sophie.mompelat@anses.fr; 4 Anses, Ploufragan-Plouzané-Niort Laboratory, Epidemiology, Health and Welfare Unit, 22440 Ploufragan, France; stephanie.bougeard@anses.fr

**Keywords:** *Salmonella*, swine, microbiota, metabolome

## Abstract

*Salmonella* is the most relevant foodborne zoonotic agent found in swine, and its presence in French herds is significant. Its carriage is asymptomatic, which makes it difficult to detect during rearing, thus increasing the risk of its presence on pork meat. Studies have shown that enteric infection in animals could be associated with changes in the serum metabolome composition, through the immune response or changes in the digestive microbiota composition. We hypothesized that these changes in the serum metabolome composition could be used as markers for the detection of asymptomatic animals infected by *Salmonella*. Using untargeted analysis by liquid chromatography coupled with mass spectrometry, we showed that significant differences in the composition of the serum metabolome could be detected between infected or noninfected animals both 1 and 21 days after experimental infection. This serum metabolome composition significantly changed during the 21 days postinfection in the infected animal groups, suggesting an evolution of the impact of infection with time. Despite this evolution, differences in the serum metabolome composition persisted between infected and noninfected animals 21 days after the initial infection. We also showed a possible difference between high-shedding and low-shedding animals 21 days postinfection. Finally, some of the variations in the metabolome were found to be significantly associated with variations of specific members of the fecal microbiota. Thus, excreting and asymptomatic animals, but also high-shedding animals, could be identified on the basis of their serum metabolome composition.

## 1. Importance

*Salmonella* is one of the most important bacterial foodborne pathogens, and salmonellosis is frequently associated with pork consumption. Better control of this pathogen at the beginning of the swine production process could help reduce the risk of infection for consumers. However, identification of infected or high-shedding swine is fastidious. Hence, new detection methods using metabolic biomarkers could be useful. The goal of this study was to evaluate the possibility of identifying these infected animals but also their level of excretion using the global serum metabolome. Our data showed that it is possible to discriminate swine infected by the monophasic variant of *Salmonella* Typhimurium using their metabolic signals both 1 and 21 days postinfection and high shedders from low shedders at 21 days postinfection. These results confirm the importance of continuing research on the impact of asymptomatic zoonotic infections on the metabolome from the perspective of identifying specific biomarkers.

## 2. Introduction

In Europe, *Salmonella enterica* is responsible for 15.7 infections per 100,000 inhabitants each year and caused 19.3% of all foodborne illness outbreaks in 2021, making this pathogen the second most reported cause of bacterially induced gastrointestinal infections and a public health priority [1]. Most infections in humans cause gastroenteritis and are contracted by consumption or mishandling of contaminated meats. Pork meat is a significant source of these cases. In Europe, it has been estimated that 31% of these cases could be attributed to its consumption [2]. 

In swine, most of the *Salmonella* infections are asymptomatic and can be carried over the whole production period in the intestinal tract, gut-associated lymphoid tissue and tonsils [3]. The presence of *Salmonella* is frequent in the French swine herds. In the last European baseline study, 39% of the French fattening farms containing breeding animals were shown to be contaminated by *Salmonella*, while the prevalence of the bacteria in the lymph nodes of the swine at the slaughterhouse was 18% [4,5]. Since it has been shown that the entrance of infected animals at the slaughterhouse is linked with a higher risk of contamination of the carcasses and hence of the infection of the consumers, better control of *Salmonella* at the beginning of the production could be beneficial in reducing the number of infections by this pathogen [6,7]. This is in line with the European strategy to optimize food safety [1]. However, identification of the infected animals or highly shedding animals in the first steps of the production is limited [3,8] because the carriage and shedding of *Salmonella* in swine are mostly asymptomatic and intermittent, and the collection of individual feces and detection of *Salmonella* by classical methods are fastidious.

Untargeted metabolomics is a powerful method that can be used to describe the global profile of the small molecules (<1 kDa) produced during the cellular processes and found in an organic sample [9]. Among the tools used to conduct these types of studies, one of the most frequently used is liquid chromatography coupled with mass spectrometry (LC–MS). Indeed, this technique has been used to describe the metabolome of multiple types of samples such as plasma, serum and feces but also to evaluate the impact of different conditions on the metabolites present in these samples [10,11]. One of the most promising applications of the metabolome study is the identification of biomarkers that could be used as diagnostic tools. Indeed, diseases, whether in animals or humans, can cause disruptions in physiological processes and activation of the immune system, leading to changes in the composition of the metabolome [12,13]. Most interestingly, multiple studies have shown the possibility of detecting specific variations in the metabolome related to these diseases but, to our knowledge, in these studies, these variations were associated with symptomatic infections [14,15]. 

One of the sources impacting the components of the metabolome is the fecal microbiota. Indeed, in the gut content, the metabolism of the bacteria that compose this microbiota is responsible for the production of multiple molecules (such as short-chain fatty acids, hormones, vitamins, etc.) that are part of, or have an impact on, the normal host metabolome [16,17,18]. In addition to the gut metabolome, it has been shown that the microbiota of the gastrointestinal tract can affect the metabolome of distant sites in the body such as the blood, kidneys and liver [19,20,21]. At least one study has shown that *Salmonella* infection can cause changes in the pig’s digestive microbiota and ileal metabolome early after infection in the presence of diarrhea and low-grade fever [22]. However, the impact of *Salmonella* infection on the overall composition of their serum metabolome and the possibility of identifying biomarkers of *Salmonella* infection in asymptomatic carrier animals have not been studied.

The first objective of this study was through an experimental trial previously conducted [23] to evaluate the possibility of discriminating asymptomatically *Salmonella*-infected pigs at 1 day and 21 days postinfection using the global serum metabolome using untargeted liquid chromatography coupled with mass spectrometry. The second objective was, on the basis of the serum metabolome composition, to assess the possibility of distinguishing low-shedder from high-shedder pigs among infected animals. Finally, we looked at variations in serum metabolome composition in relation to variations in the fecal microbiota of the corresponding animals.

## 3. Material and Methods

### 3.1. Animal Procedures

As described in Kempf et al., 2023 [23], this trial was conducted on 45 specific pathogen-free Large White piglets born from 5 sows at the Anses Ploufragan’s protected animal facilities. These piglets were weaned at 4 weeks of age and distributed in 5 separate units containing 1 pen of 5 animals for the controls and 2 pens of 5 animals each for the infected groups. Piglets were distributed following a random-cluster criteria balancing by sex and weight and, to avoid maternal effect, 1 piglet born from each sow was present in each pen. Animals were fed ad libitum using manual feeders with a standardized diet and had free access to water through automatic drinking fountains.

At 7 weeks of age, the animals were infected with 10^8^ CFU of a rifampicin-resistant monophasic variant of *Salmonella* Typhimurium (mST) strain, while 5 animals were inoculated with a tryptone salt solution as control [23]. Following infection, individual feces and blood samples were collected at 1, 7, 14 and 21 days postinfection (DPI). For each of the collected fecal samples, a subsample was frozen at −80 °C for microbiota analysis, while *Salmonella* counts were conducted on the individual fresh feces using XLD media (Biokar, Allonne, France) supplemented with rifampicin (100 mg/l, Sigma, Saint-Quentin-Fallavier, France). Blood samples were collected in 9 mL vacutainer tubes without any additives (Becton Dickinson, Le Pont de Claix, France) and sera were obtained by centrifugation at 3500× *g* for 5 min before freezing. The seropositivity of the animals was tested on the serum by measuring the level of *Salmonella* antibodies using IDEXX Swine *Salmonella Ab* Test ^®^ (IDEXX, Saint-Denis, France) according to the manufacturer’s recommendations. The in vivo trial protocol was approved by the Ethics Committee on Animal Research No. 16 of the French Ministry of Research (APAFiS license No. 16570-2018083015249240). 

In the previously mentioned study, the infected swine were classified into three groups (high, intermediate and low shedders) using the area under the curve of their *Salmonella* shedding kinetics through the 21 days of the trial [23]. This same classification was used in this study; however, the intermediate shedders were removed from the dataset. The samples collected 1 and 21 days postinfection from the 13 animals identified as high shedders (HS) and from the 11 animals identified as low shedders (LS) were used for our study, as well as the 5 noninfected (NI) animals used as controls in the trial for a total of 58 sera and fecal samples. 

The only symptom observed following the infection was a significant increase in the body temperature of the 40 swine in the inoculated group of 0.6 °C at 1 DPI without significant differences according to shedding levels [23].

### 3.2. Metabolomic Analysis

#### 3.2.1. Samples Preparation

Metabolites were extracted by the addition of 100 µL of serum to 500 µL of a cold (−20 °C) methanol (Fisher Scientific, Illkirch, France) and acetonitrile (Fisher Scientific, Illkirch, France) mixture solution (50/50 *v*/*v*). Samples were vortexed for 30 s and kept at −20 °C for 30 min. They were then centrifuged at 15,000× *g* for 10 min at 4 °C, and the supernatants were transferred for concentration in a TurboVap (Biotage, Uppsala, Sweden) evaporator at 30 °C under a continuous flow of nitrogen until complete evaporation. The extracts were reconstituted with 100 µL of water containing 0.1% of formic acid 98/2 (*v*/*v*) (Supelco, Saint-Quentin-Fallavier, France)/methanol. Then, 50 µL of a solution containing 9 isotopically labeled internal standards was added to each sample, and the samples were filtered at 0.22 µm before being transferred to an LC–MS conical vial. Quality control samples (QC) containing the whole library of metabolites found in the samples were also prepared by pooling 10 µL of each sample extract.

#### 3.2.2. LC–MS

Metabolomic data acquisition was carried out by high-performance liquid chromatography coupled with high-resolution mass spectrometry (HPLC–HRMS) with an electrospray ionization source operated in both negative and positive ionization modes. Separation was conducted on an ultra-high-performance liquid chromatography (U-HPLC) Vanquish Flex (Thermo Fischer Scientific, Illkirch, France) using an Accucore Phenyl-Hexyl column (100 mm length × 2.1 mm diameter, 2.6 µm particle size, Thermo Scientific, Illkirch, France), during 30 min at a flow of 300 µL/min and at a temperature of 30 °C. The gradient of the mobile phase ranged from 2% methanol (Optima ^®^, LC–MS grade, Fisher Scientific, Illkirch, France) in water containing 0.1% formic acid (Supelco, Saint-Quentin-Fallavier, France) to 98% methanol. Mass spectrometry detection was conducted on a Q Exactive™ Plus Hybrid Quadrupole-Orbitrap™ Mass Spectrometer (Thermo Fisher Scientific, Illkirch, France). Note that 5 µL of each sample was injected in a random order, and one QC sample was injected for every five samples in order to monitor the analytical system variability. Two blank samples containing all the reagents used in the extraction without the serum were also injected at the beginning and the end of the experiment. Spectra were acquired in full scan mode for all samples.

#### 3.2.3. Data Extraction and Metabolic Signals Identification

Data preprocessing was conducted using the XCMS package version 3.12.0 [24] on the workflow4metabolomics (W4M) galaxy platform [25]. Chromatographic peak detection was conducted using the centWave method of the findChromPeaks function. For this step, the maximum tolerated ppm *m*/*z* deviation in consecutive scans was set to 15, the minimum and maximum peak widths to 10 and 60 s, respectively, and the minimum difference in *m*/*z* for peaks with overlapping retention times of 0.015. All the other settings were used with their default values. Peaks grouping was conducted using the peak density method of the groupChromPeaks function using a bandwidth setting of 5, while the other parameters used standard values. Retention time variation was corrected using the PeakGroups method of the adjustRtime function with default settings. After retention time correction, a second peak grouping step was conducted using identical settings. Finally, missing peaks were filled using the fillChromPeaks function. After these steps, extracted ion chromatograms were created for each of the standards to ensure that the selected setting correctly detected these features.

The CAMERA (Collection of Algorithms for MEtabolite pRofile Annotation) package was then used for the annotation of isotope peaks, adducts and fragments in peak lists.

Signal drift was corrected using the Batch_correction function of the workflow4metabolomics pipeline using the all loess pool method. Using this method, a regression model is fitted to the values of the pools (QC samples) and used to adjust the intensity of the metabolic signals in the samples [26].

Features for which signal intensity value could not be obtained (NA) for all samples were removed. Those that had a coefficient of variation of more than 30% in the QC samples were also removed as the features that had an intensity in the QC samples lower than 3 times the intensity in the blank samples. Metabolites Correlation Analysis was conducted to identify correlated features in the different peak cluster groups corresponding to different ions of the same molecule. The features with the highest intensity were kept, while the others were discarded. 

The samples were compared using Hotelling’s t-squared distance and intensity distribution decile Z-score to detect outliers and removed from the dataset when at least one of these statistical tests was highly significant (*p* < 0.001). For the positive acquisition mode, two samples from the low-shedder group were removed, one from the animals 1 DPI and one 21 DPI, and for the negative acquisition mode, one sample from the high-shedder 21 DPI group.

Finally, the intensity of the metabolic signals in each sample was normalized using the total intensity method of W4M.

### 3.3. Microbiota Analysis

#### Samples Preparation

Extraction of total DNA was conducted as explained in Kempf et al., 2023 [23], and sequencing was conducted by The Earlham Institute (UK) on an Illumina MiSeq platform obtaining 300 bp paired ends raw reads. Raw sequences are available on the Dryad data repository (https://doi.org/10.5061/dryad.wwpzgmsm3 (accessed on 8 June 2023)).

The raw sequences were treated using Mothur v 1.45.2 [27] following an adapted version of Mothur’s standard operation procedure, as described in Larivière-Gauthier et al., 2017. Unique sequences were aligned using the Mothur-adapted version of the SILVA database (silva.seed.v132, https://mothur.org/wiki/silva_reference_files (accessed on 8 June 2023)) [28]. After the preclustering step, singleton sequences were removed. The remaining sequences were grouped in an operational taxonomic unit with 97% similarity (OTU). Those OTUs were attributed a taxonomic classification using the Mothur-formatted Ribosome Database Project (RDP) trainset version 18 (https://mothur.org/wiki/rdp_reference_files/ (accessed on 8 June 2023)). 

### 3.4. Statistical Analysis

#### 3.4.1. Serum Metabolome Analysis

The effects of the different conditions on the composition and intensity of the metabolomic signals found in the serum were compared between groups, initially by visualization using principal component analysis (PCA) and 95% ellipses. To confirm differences visualized on the PCA graphs, partial least square discriminant analysis (PLS-DA) models were built. The differences between the compared groups were considered significant when: (i) the R^2^Y and Q^2^ values for the model were of similar amplitude and high (over 0.700), and (ii) the permutation tests conducted on each of these values were significant (*p* < 0.05). Variables that mainly influenced the discrimination of the groups in these models were extracted using the Variable Importance in Projection scores (VIP). This score estimates the importance of each variable in the projection used in a PLS model. A variable with a score greater than 1 is considered important (VIP score > 1). All the analyses were conducted using the ropls version 1.32.0 R package [29]: opls function with the orthoI (number of orthogonal components) option set to 0 for PLS-DA, getVipVn function for the VIP. 

#### 3.4.2. Fecal Microbiota Analysis

To compare the effect of the different conditions on the diversity of the OTUs found in each individual feces sample, alpha diversity indices were calculated. First, the number of sequences in each sample was randomly rarefied to the lowest number of sequences in a sample to eliminate the bias caused by samples with higher sequencing depth. Then, the number of observed OTUs, the Shannon evenness and the inverted Simpson’s indices were measured. This procedure was repeated 100 times, and the average value obtained for each index was calculated to reduce the impact of possible outlier results that could be caused by the random rarefaction. The normality of the data was not respected. Hence, these indices were compared using the Mann–Whitney test for comparisons between 2 groups, while for 3 groups, the Kruskal–Wallis test followed by Dunn’s post hoc test with Benjamini–Hochberg false discovery rate correction for pairwise comparisons was used. The rarefaction was conducted using the rarefy_even_depth function, and alpha indices were calculated using the estimate_richness function, both from the phyloseq package version 1.34.0 under R [30].

To measure the impact of the different conditions on the structure and composition of the feces microbiota, beta diversity analysis that quantifies the distance between samples was conducted. After rarefaction, the dissimilarity between each pair of samples was calculated using the Bray–Curtis distance, which compares samples on the basis of the composition of OTUs and their abundance. A distance matrix was produced. This procedure was repeated 100 times with a new rarefaction at each iteration, and a matrix containing the average distance values was calculated to reduce the impact of possible outlier rarefaction results. The beta diversity of the different groups was statistically compared by PERMANOVA with a significance level of 0.05 and for pairwise comparisons, the *p*-value was adjusted using false discovery rate correction. Distance matrices were produced using the avgdist function, and PERMANOVA tests were conducted using the ADONIS2 function, both from the vegan 2.6–4 package under R [31].

#### 3.4.3. Link between Fecal Microbiota Composition and Serum Metabolome Composition 

Changes in fecal microbiota in the different groups that could explain variations in serum metabolic signals were explored. These links were investigated when significant global differences were found between the two conditions for both sets of data (microbiota and metabolome). First, variables that had a significant variation in the compared groups with a *p*-value of 0.05 for the OTUs and 0.005 for the metabolic signals were selected. Regression analysis models were then built using a sparse partial least square method, which reduces the number of variables with a LASSO penalization on both datasets. A link between an OTU and a metabolic signal variation was considered important when a positive association (higher than 0.5) or negative association (lower than −0.5) was obtained in the final model. These analyses were conducted with the mixOmics 6.19.1 package under R [32] using the perf function to select the optimal number of components, the tune.spls function to select the optimal number of variables and the spls function to build the final models.

### 3.5. Data Availability

The raw sequencing dataset is available on the Dryad repository https://doi.org/10.5061/dryad.wwpzgmsm3 (accessed on 8 June 2023).

## 4. Results

### 4.1. Serum Metabolome Analysis

After removing outlier samples, of the 58 serums selected from the sampled animals, 57 samples of serum were kept for data analysis for the negative acquisition mode and 56 for the positive mode. After cleaning of the LC–MS data, a total of 3041 high-quality metabolic signals were retained in positive acquisition mode and 2136 in negative acquisition mode. 

### 4.2. Comparison of Infected Pigs to Noninfected Animals

The serum metabolome of the infected animals (HS and LS, 24 animals) was first compared with that of the NI animals (5 animals) at both 1 DPI when shedding was near its maximum (5.09 +/− 1.65 Log10 CFU/g) and at 21 DPI when shedding was decreased (3.75 +/− 1.67 Log10 CFU/g).

**Figure 1:**
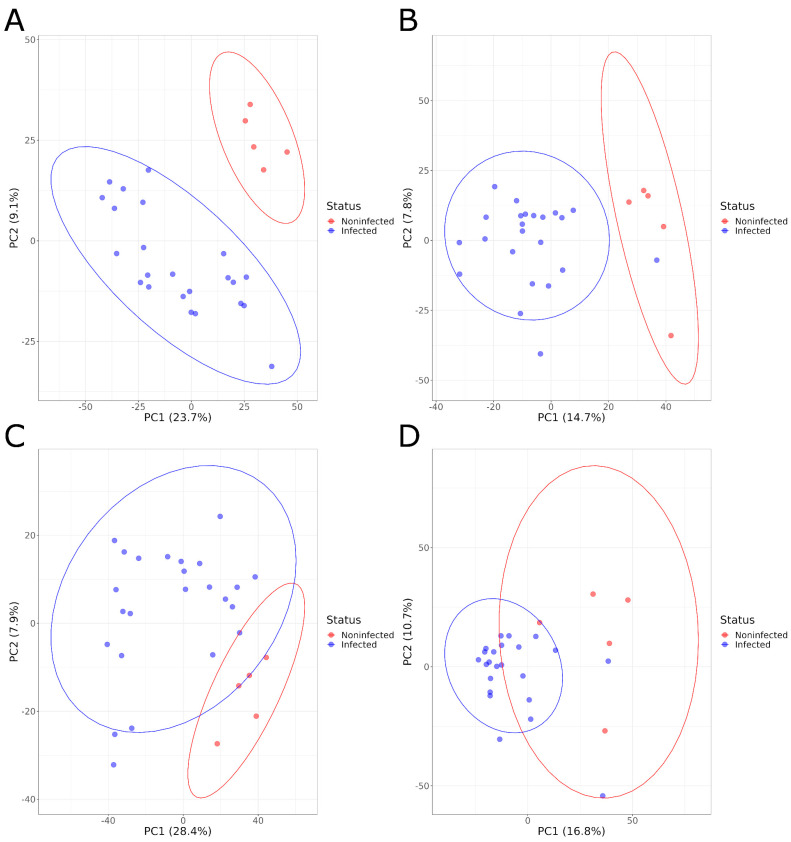
PCA graphs comparing the serum metabolome composition of infected and noninfected animals in positive acquisition mode (**A**) 1 DPI and (**B**) 21 DPI and negative acquisition mode (**C**) 1 DPI and (**D**) 21 DPI.

PCA plots showed a clear separation of serum samples from infected animals and NI animals at both sampling times and for both positive and negative ionizations, based on the overall serum metabolome composition (Figure 1). The PCA results were confirmed by the PLS-DA models that were built with those samples. These models showed a high R2Y value, indicating the ability of the model to explain a large fraction of the variance for the training samples, and a high Q2, showing that the model can achieve, using test samples, a good classification of infected and noninfected animals. The statistical significance of these two parameters was confirmed by the permutation test at both 1 and 21 DPI for the negative ionization mode, but only at 1 DPI for the positive mode with a non-significant pRY2 value (*p* > 0.098) at 21 DPI (Table 1, Supplementary Figures S1 and S2). 

For models constructed using the negative acquisition mode, 1065 and 1100 metabolic signals had a VIP score greater than 1 at 1 DPI and 21 DPI, respectively. Of those metabolic signals, 413 were important at both sampling dates. For the single significant model constructed with data in the positive acquisition mode at 1 DPI, 1046 metabolic signals were considered to be important (VIP score > 1).

### 4.3. Comparison According to the Shedding Levels of SALMONELLA

The metabolic signals were also analyzed by considering the three groups of animals (HS, LS and NI animals). PCA graphs showed a good separation between the samples from *Salmonella* shedder animals and the samples from NI animals. 

**Figure 2:**
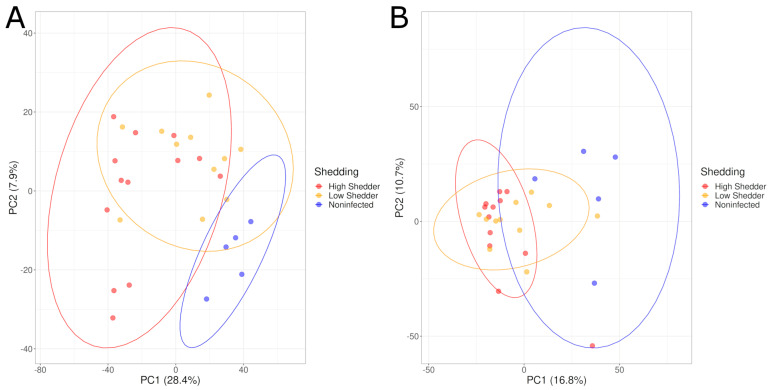
PCA graphs comparing the serum metabolome composition of low shedders, high shedders and noninfected animals in negative acquisition mode (**A**) 1 DPI and (**B**) 21 DPI.

However, the separation between HS and LS was not clearly apparent (Figure 2). PLS-DA models showed high values for the performance indicators, with significant permutation tests indicating significant differences when considering these three groups for the negative acquisition mode at 1 DPI and at 21 DPI. For the positive acquisition mode, the model produced using the samples collected at 1 DPI also showed good discrimination between these groups with both high R2Y and Q2 values and significant permutation tests. However, no significant difference was observed for the samples at 21 DPI because the model showed a low Q2 value of 0.419, indicating inadequate classification performance (Table 1, Supplementary Figures S3 and S4).

**Figure 3:**
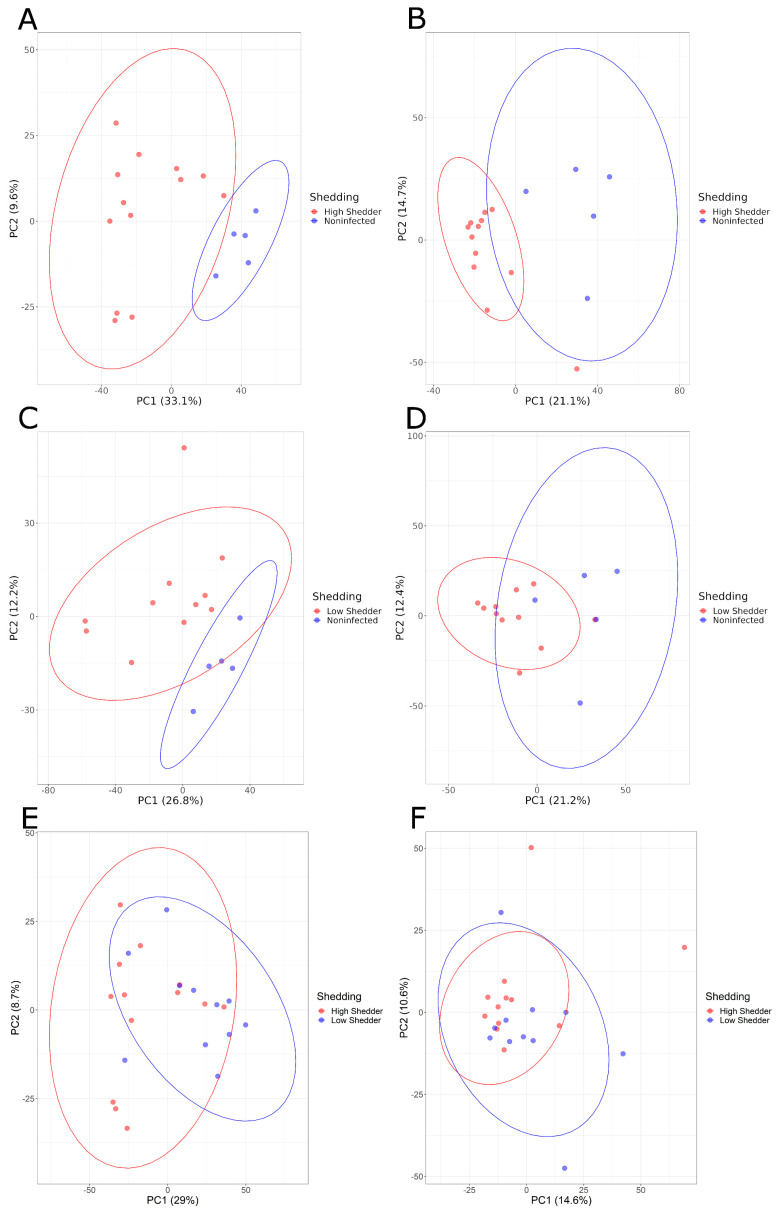
PCA graphs of pairwise comparisons of the serum metabolome composition of low shedders, high shedders and noninfected animals in negative acquisition mode (**A**,**C**,**E**) 1 DPI and (**B**,**D**,**F**) 21 DPI.

Pairwise comparisons of these three groups were conducted. PCA graphs (Figure 3) showed a good separation between HS and NI animals, and between LS and NI animals both at 1 and 21 DPI, and for both the negative and positive ionization modes. These differences were confirmed by PLS-DA models with a high-performance indicator and a significant permutation test. For the comparison between HS and LS, no clear separation could be visualized on the PCA (Figure 3E,F). However, while no model matching the performance criteria could be produced for the positive ionization data, a model showing a high discrimination performance was, however, obtained for the negative ionization at 21 DPI (Table 1, Supplementary Figures S5 and S6).

For models constructed using the positive acquisition mode, when discriminating HS versus NI animals, 1188 and 1127 metabolic signals had a VIP score greater than 1 and were therefore considered important in the discrimination models at 1 DPI and 21 DPI, respectively. Moreover, 417 of those metabolic signals were important at both sampling dates. Comparing LS and NI animals, 1022 and 1102 metabolic signals were identified as important at 1 DPI and 21 DPI, respectively, with 408 common metabolic signals. At 1 DPI and at 21 DPI, 661 and 721 metabolic signals were common between the HS and LS, respectively, with 724 common metabolic signals. Finally, of all those metabolic signals, 163 were found to be important at both dates and both for the discrimination of HS and LS from the NI animals.

For the discrimination models obtained in the negative acquisition mode, 1149 metabolic signals at 1 DPI and 1088 at 21 DPI were considered important (VIP > 1) for the classification of the HS and NI animals. These metabolic signals represent those that are most affected by the infection. Of these metabolic signals, 426 were important at both dates. When comparing LS and NI animals, 1059 and 1159 signals were important at 1 DPI and 21 DPI, respectively, with 412 common metabolic signals. 

At 1 DPI and 21 DPI, 592 and 726 of these important metabolic signals were, respectively, common to both the model comparing HS to NI animals and the one comparing LS to NI animals. Finally, 162 metabolic signals were important for the models both for the HS and LS at both dates of sampling. 

### 4.4. Comparison According to Time Postinfection

**Figure 4:**
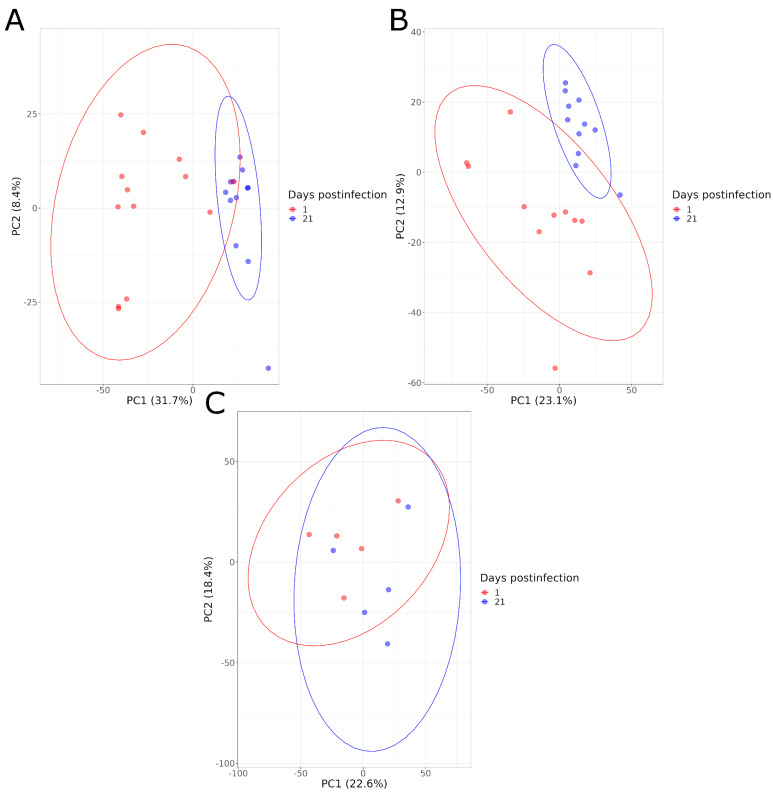
PCA graphs comparing the serum metabolome composition of animals at 1 and 21 DPI in negative acquisition mode for high shedders (**A**), low shedders (**B**) and noninfected animals (**C**).

The serum metabolome composition of animals in each group (HS, LS, NI) was compared at 1 and 21 DPI. The PCA graphs illustrated a good separation between these two time points for the infected animals, for both levels of shedding, and both for the negative and positive ionization modes. This is not the case for the NI animals (Figure 4). A higher distance between animals within infected groups (HS and LS) was also observed at 1 DPI compared to 21 DPI, indicating a greater heterogeneity of the metabolome composition at the beginning of the infection (Figure 4).

These results were also confirmed by the PLS-DA models that showed high-performance parameter values and significant permutation tests. This indicates the ability of the models to discriminate the samples for the infected animals, while the models had a poor performance for the NI animals with Q2 values under 0.6 and non-significant permutation tests (*p* > 0.05), showing no significant differences in this group (Table 2, Supplementary Figures S7 and S8). 

For the positive acquisition mode, at both 1 DPI and 21 DPI, 1250 metabolic signals were important in the PLS-DA models (VIP > 1) for the discrimination of animals for HS, and 978 for LS, with 588 signals in common between the two groups. For the negative acquisition mode, 1151 and 1050 metabolic signals were important for HS animals and LS, respectively, with 613 metabolic signals in common. 

### 4.5. Comparison According to the Salmonella Seropositivity of the Animals

At 1 DPI, no seropositivity for *Salmonella* was measured in animals infected or not, while at 21 DPI, 8 of the 13 HS and 6 of the 11 LS were *Salmonella*-seropositive while the noninfected animals stayed seronegative. 

The serum metabolome composition of the animals was compared on the basis of their seropositivity both at 21 DPI comparing the seropositive animals to the seronegative animals but also at 1 DPI comparing the animals that have become seropositive during the trial to those that stayed seronegative until the end of the trial. PCA graphs showed no clear separations between the two groups, seropositive animals and seronegative animals (Supplementary Figure S9). However, for the metabolomic data acquired in positive mode, the PLS-DA models showed significant differences in the serum metabolome composition of animals at 1 DPI when comparing the totality of the animals that would become seropositive and seronegative animals at 21 DPI without distinction of shedding groups (Table 3, Supplementary Figures S10 and S11). In this model, 1275 metabolic signals were considered important for the discrimination (VIP values > 1).

### 4.6. Fecal Microbiota Analysis

Of the 58 animals included in this study, 57 fecal samples were sequenced (one sample from an HS at 21 DPI could not be collected). After processing the raw data using the Mothur software version 1.46.0 to remove sequencing and PCR errors, 1,622,991 sequences with good quality were kept and formed 2869 OTUs. The average number of sequences per sample was 28,473 for 489 OTUs, with a maximum of 56,970 (627 OTUs) and a minimum of 15,111 (233 OTUs).

#### 4.6.1. Comparison According to the Shedding Levels of *Salmonella*

The alpha diversity indices calculated after rarefaction were compared between the microbiota of the animals that were infected and noninfected at both days after infection. No significant difference between these two groups was observed for the Shannon index, inverted Simpson’s index, and evenness (*p* > 0.05). However, for both sampling dates, the number of observed OTUs was significantly higher in the NI group (Mann–Whitney test 1 DPI *p* = 0.03, 21 DPI *p* = 0.01). When considering the groups HS, LS and NI animals, a significant difference could be measured for the number of OTUs both at 1 and 21 DPI (Kruskal–Wallis test *p* = 0.03, *p* = 0.03). Pairwise comparisons at 1 DPI showed a significant difference between the NI animals and the LS animals and at 21 DPI between the HS and NI (Dunn’s test LS/NI 1 DPI *p* = 0.02, HS/NI 21 DPI *p* = 0.03). However, no significant difference between HS and LS was observed (Dunn’s test *p* > 0.05) (Table 4).

Beta diversity was compared using the PERMANOVA statistical test on Bray–Curtis dissimilarity matrices (Table 5). A significant difference in the composition of the fecal microbiota was observed when comparing the infected animals to the NI animals, both at 1 and 21 DPI (PERMANOVA 1 DPI *p* < 0.037, 21 DPI *p* < 0.018). However, no significant difference was observed between HS and LS (PERMANOVA *p* > 0.05). Still, the pairwise comparison showed significant differences between both HS and LS and NI animals at 21 DPI (PERMANOVA HS/NI *p* < 0.048, LS/NI *p* < 0.048); no significant difference was observed at 1 DPI, as well as for the comparisons between the HS and LS animals after false discovery rate correction (PERMANOVA *p* > 0.05).

#### 4.6.2. Comparison According to Time Postinfection

For the alpha diversity comparison between animals at 1 DPI and 21 DPI, all the calculated indices were significantly higher at 21 DPI for the infected animals (HS and LS) but also for the noninfected animals (Mann–Whitney *p* < 0.05) (Table 6). 

The global composition of the fecal microbiota of the animals was also compared between 1 DPI and 21 DPI for all the shedding statuses. Significant differences were observed between animals at 1 and 21 DPI for the two shedding levels (HS and LS) but also for the noninfected animals (PERMANOVA *p* < 0.05) (Table 7).

#### 4.6.3. Comparison According to the *Salmonella* Seropositivity of the Animals

The composition of the fecal microbiota of the infected animals was compared on the basis of their seropositivity for *Salmonella*. No significant difference was observed both for the alpha and beta diversity when comparing the seropositive to the seronegative animals at 21 DPI or at 1 DPI when comparing the animals that would become seropositive later at the end of the trial to those that would stay seronegative (Mann–Whitney *p* > 0.05, PERMANOVA *p* > 0.05) (Table 8 and Table 9). 

### 4.7. Link between Fecal Microbiota Composition and Serum Metabolome Composition

As we measured significant differences in the serum metabolome composition and in the fecal microbiota composition in this study, we explored the possibility of a link between variations in bacterial fecal populations and changes in the serum metabolome composition. First, statistical links between variations in the microbiota (represented by the number of sequences for each OTU) and the metabolome (represented by the intensity of the metabolic signals) were found between infected and noninfected animals. These links could be observed whatever the acquisition mode considered for metabolomic data. 

For the positive acquisition mode, the variation of intensity measurements for 127 and 76 metabolic signals were found to be linked with the variation of the number of sequences for 5 OTUs and 14 OTUs, at 1 DPI and 21 DPI, respectively. In both cases, the results showed that the OTUs were each linked with multiple metabolic signals, while metabolic signals could also be linked with multiple OTUs. Six metabolic signals and one OTU were common between those identified at 1 DPI and 21 DPI. However, for the same OTU/metabolic signal association, none of the links relating to these variables had the same direction (ex.: OTU positively or negatively associated with a metabolic signal) at the two sampling points (Supplementary Tables S1 and S2, Supplementary Figure S12). 

For the negative acquisition mode, 103 metabolic signals and 4 OTUs at 1 DPI and 74 metabolic signals and 15 OTUs at 21 DPI were found to be significantly associated; once again, with OTUs linked to multiple metabolic signals and metabolic signals linked to multiple OTUs. No OTUs involved in these associations were common at 1 DPI and 21 DPI, while 11 common metabolic signals were found implicated in the association at 1-DPI and 21 DPI. At 1 DPI, of the 5 OTUs that had a significant association with a metabolic signal in the positive acquisition mode, one was also significantly associated in the negative mode, while at 21 DPI, 12 of the 15 OTUs highlighted in the negative mode play this role also in the positive mode (Supplementary Tables S3 and S4, Supplementary Figure S12). 

When considering the three groups (HS, LS, NI), the sPLS analyses were only conducted when both the metabolome and the microbiota of the compared groups were shown to be significantly different, limiting this analysis to the comparison between HS and NI animals and between LS and NI animals at 21 DPI. For HS and NI animals, comparison in the negative acquisition and positive modes showed, respectively, that the changes for 216 and 259 metabolic signals were linked with 25 OTUs and 32 OTUs, respectively, with 23 OTUs common to both acquisition modes. For LS and NI animals, 127 metabolic signals were linked with 34 OTUs for the negative mode and 106 metabolite signals with 41 OTUs in the positive mode. Twenty-eight of these OTUs were common to both acquisition modes (Supplementary Tables S5–S8, Supplementary Figure S13). 

Finally, the association of the variation of the microbiota composition and the serum metabolome composition was explored when comparing the infected animals at 1 DPI to animals at 21 DPI. For HS, the variations of 160 OTUs and of 228 OTUs were linked to at least one of the 909 metabolic signals and to at least one of the 881 metabolic signals, in positive and negative acquisition mode, respectively. The 160 OTUs that were found to be linked in the positive acquisition mode were among the 228 associated with the negative mode. For the LS, the variations of 55 OTUs and 118 OTUs were linked to 394 and 314 metabolic signals, in positive and negative acquisition mode, respectively. Similarly to the results for HS, the 55 OTUs that were found to be linked in the positive acquisition mode were all found to be linked in the negative acquisition mode (Supplementary Tables S9–S12, Supplementary Figure S14). 

For the positive acquisition mode, 55 OTUs and 305 metabolic signals were found to have significant associations for both the HS and LS groups, and for 83.5% of these associations, the direction of the interaction was the same for both groups, while for the negative acquisition mode, 114 OTUs and 224 metabolic signals were common and 95.6% of the associations had the same direction for the HS and LS groups (Supplementary Tables S9–S12, Supplementary Figure S14). 

## 5. Discussion

In this study, we investigated the possibility of differentiating monophasic variant *Salmonella* Typhimurium-infected pigs from negative pigs but also -infected pigs with different levels of shedding using the global metabolome composition of the serum of experimentally infected pigs. We also evaluated the possible link between changes in the serum metabolome composition and variations in the fecal microbiota composition.

### 5.1. Comparison of Infected Pigs to Noninfected Animals

First, we evaluated the effect of this *Salmonella* infection on both the metabolome and the microbiota of the animals.The results obtained here show that the infected animals have a significantly different metabolome from the noninfected animals. Indeed, it appears that the infection by mST is associated with changes in the global metabolome composition of the animals and that it is possible, using PLS-DA models, to discriminate infected animals from noninfected animals as soon as 1 day after the infection. Interestingly, significant PLS-DA models could also be generated 21 days after the infection when the infection is completely asymptomatic, however, only for the negative acquisition mode and with lower Q2 values. These results show that the animals could still be discriminated, albeit with a lower accuracy, even multiple days after the infection in the absence of symptoms. This suggests that in swine the asymptomatic carriage of mST induces permanent or at least long-term changes in the metabolome of the animals. Furthermore, an important part of the metabolic signals that were important for the discrimination of the two groups by the PLS-DA model were common at the two sampling dates. This shows that at least a part of the metabolic differences measured between the infected and noninfected pigs 1 day postinfection is kept during the 21 days post infection. Further experiments are needed to confirm that these changes are still present beyond day 21 after infection. It also seems that in the context of our experiment, even if the positive mode of acquisition in HRMS resulted in a higher number of metabolic signals, those detected in the negative ionization mode had a higher discriminant power at the 21 days post infection. 

To the authors’ knowledge, there are currently no studies on the effect of *Salmonella* on the overall serum metabolome of pigs, both early and late after the infection. However, a previous study conducted on swine infected by *Salmonella* showed significant modification of the metabolic composition of the ileum mucosa with a reduction of the basal metabolism two days post infection possibly linked with modification of the microbiota [23]. Similarly, metabolic changes were measured in the cecal metabolome of chickens infected by *Salmonella* up to 14 days after infection. These authors emitted the hypothesis that persistent infection of these animals by *Salmonella* triggers immunometabolic changes in the host in order to limit cellular damage [33]. Indeed, metabolic alterations that occur in the cecal tissues could transition from the early resistance phase, which is proinflammatory, to a later tolerance phase, in which the local cecal environment undergoes a transition to an anti-inflammatory state, and finally to a homeostasis phase of disease tolerance [34]. Studies showed that infection by *Salmonella* Typhimurium can cause changes in the chicken muscle skeletal metabolism and that infection by the parasite *Cryptosporidium baileyi* was linked with variation of the serum metabolome [35,36]. Like our results, these studies indicate that alterations caused by enteric pathogens infection are not restricted to the digestive system. However, our results are the first to show that the overall metabolic fingerprint of serum is modified in mST-infected pigs, even multiple weeks after infection, under conditions where the animals are asymptomatic. 

In addition, changes in the serum metabolome could also be caused by changes in the composition of the animals’ gut microbiota after infection. Indeed, it has been shown in multiple recent studies that the fecal microbiota is linked with the serum metabolome [37,38,39]. As we showed in this study, infected animals had a significantly different microbiota than noninfected animals at both sampling time. It is possible that some of the serum metabolomic changes measured here are the result of variations in metabolites produced by the microbiota in the animal’s digestive system after infection. Indeed, using sPLS analysis, we showed that some of the bacterial OTUs that had significantly different levels of presence in infected versus noninfected animals were associated with changes in metabolic signals. Interestingly, some of the OTUs were associated with multiple metabolic signal modifications and both for the positive and negative acquisition mode. This suggests that one taxa of bacteria could influence multiple metabolites found in the serum. However, none of these identified OTUs were individually linked with metabolic signals variations both at 1 and 21 days postinfection. The number of metabolic signals that were found to be associated with the microbiota when comparing infected to noninfected animals appears low compared to the high number of metabolic signals that were considered important for building a model discriminating these two groups. This suggests that for the metabolic variations caused by the infection by the monophasic variant of *S. typhimurium*, the impact of the microbiota could be limited. 

### 5.2. Comparison According to the Shedding Levels of Salmonella

As specified previously, infected animals considered in our study could be distributed in two groups of different shedding levels, high- and low-shedders. Previous studies have suggested that these differences could be explained by multiple factors such as health status, immune response, microbiota composition or function and animal age [40,41]. Interestingly, all these factors could affect the serum metabolome of the animals. In our study, the results obtained for the metabolomic comparisons showed that both low shedding and high shedding animals could be discriminated from the noninfected animals. At one-day postinfection, significant differences could be measured in the serum metabolome composition even in the absence of a significant microbiota composition modification. This is compatible with a quick physiological response following infection while microbiota is impacted later in the trial course. Furthermore, significant impact on the metabolome were also measured for the animals that have low levels of *Salmonella* shedding. However, when comparing the two infected groups (high shedders and low shedders) they could not be differentiated except at day 21 postinfection for the negative mode of acquisition. These mixed results seem to indicate that, shortly after the infection, the metabolome of the high and low shedding animals are globally similarly impacted by the introduction of the pathogen and could evolve differently during the course of the infection. These differences of the metabolome do not seem to be linked with strong variation in the overall microbiota composition since no significant differences in its composition were measured between these two groups. This seems to confirm the other results obtained in this experiment showing that most of the modifications of the metabolome following infection by mST could not be linked with variations of the fecal microbiota. In the context of our experiment, conditions are not met to conclude whether the measured differences in the metabolome are involved in the different levels of excretion or whether these different levels of excretion cause different changes in the metabolome, or both. Further studies describing the serum metabolome of swine before infection and a link be-tween the evolution of these metabolomes in association with multiple excretion levels could be a first step in resolving this question.

### 5.3. Comparison According to Time Postinfection

Following these results, we evaluated the evolution of the metabolome composition and the fecal microbiota composition of our animals between the first day of the experiment (1 day post infection) and three weeks later (21 days postinfection).

Our results showed significant differences in the metabolome of the infected animals between day 1 post infection and day 21 for both levels of shedding, while no differences could be detected for the noninfected animals. Furthermore, for the two infected groups, a greater variability in the metabolome composition was observed one day post infection than 21 days postinfection. The fact that no changes could be measured for noninfected animals suggests that the overall variations observed for infected animals are consistent with a host response to mST rather than a physiological evolution of the metabolome, which might be expected from maturation of the animal. Similar results were obtained in other studies where evolution of the serum metabolome composition was observed over time after infection. For example, in swine, infection with *Mycoplasma hyopneumoniae* has been shown to affect serum levels of certain amino and fatty acids in a time-dependent manner [42]. The greater variability of the metabolome shortly after mST infection suggests a metabolic destabilization or heterogeneous response of the organism that attenuates over time. These changes could be associated with a transition from a lightly symptomatic infection 1 day after infection to a completely asymptomatic carriage of the pathogen at 21 days. Indeed, the slight increase in temperature observed in infected pigs on the first day post infection could be the result of an inflammatory reaction caused by the infection that subsides with time. Interestingly, even with this evolution of the metabolome during the infection, significant differences could still be measured between infected and noninfected animals at day 21. As mentioned previously, we hypothesized that these changes could be the result of an evolution of the immune response with a reduction in inflammation from the beginning of the infection to the end of the experiment, but could also be related to an evolution of the fecal microbiota. Indeed, the microbiota of these animals was significantly different in terms of alpha and beta diversity between the two time points whether the pigs were infected or not. A significant difference in the composition of the fecal microbiota was also measured for the noninfected animals, so that part of the evolution of the microbiota during the experimental period occurred independently of mSTinfection, and this evolution was not associated with a change in the overall metabolome in these noninfected animals. An evolution of the fecal microbiota over the lifetime of the animal has already been described, with similar changes in alpha diversity indices showing an increase in the complexity of the fecal microbiota over time [43,44]. As explained earlier, some studies have shown that *Salmonella* infection can affect the fecal microbiota of swine. Therefore, we cannot exclude that some of the differences measured here in the infected groups are associated with the infection, in addition to those caused by normal maturation of the microbiota, and that these changes may also have an impact on the serum metabolome composition. Interestingly, a higher number of metabolic variations were associated with changes in fecal microbiota for the temporal comparison than for the comparison of the shedding status of the animals. Most of these significantly linked variations were found in low shedding animals and high shedding animals with the same direction. This seems to show that the metabolic variations measured between one day postinfection and 21 days postinfection are driven by variations of the microbiota.

### 5.4. Comparison According to the Salmonella Seropositivity of the Animals

Finally, in this study, only a portion of the infected animals were seropositive after 21 days. Studies have shown that the dynamic of the animal seroconversion is variable and appears to be related to farm practices that primarily affect the time or level of exposure to the pathogen [45,46,47]. However, in the case of our study, these factors could not explain the difference in seroconversion between animals since all pigs were infected at the same time with the same dose of inoculum and were reared under the same conditions in the animal facility. Differences in the microbiota or the metabolome compositions were therefore explored as an explanation. No changes in either metabolome or microbiota at 21 days post infection could be linked to seropositivity. Thus, the measured changes in animal serum metabolomes occurred independently of changes in the serostatus of the pigs. However, for the positive ionization mode, it was possible 1 day post infection to distinguish animals that would become seropositive during the course of the experiment from those that would remain seronegative using the overall metabolome composition. It might be interesting to see if these results indicate that animals that become seropositive have a different initial immune response to mST infection, which might lead, for example, to faster antibody production later in the course of infection. However, in the case of our experiment, it is not possible to know whether these differences were already apparent prior to infection or whether they emerged following infection. Furthermore, we did not measure any significant difference in fecal microbiota taking into account the serology of the animals. Therefore, it does not appear that differences measured here in the metabolome composition are related to overall variations of fecal microbiota.

One weakness of this study is the lack of comparison of the metabolic response to another similar pathogen. Indeed, in the context of this experiment, it is not possible to conclude whether the measured variations in metabolic signals are specific to a *Salmonella* infection or whether they correspond to a generic response to infection with an enteric bacterial. Hence, it’s not possible to conclude with certainty that this method can identify animals specifically infected by *Salmonella*. Further experiments comparing the serum metabolome of pigs infected with different pathogens both relevant in food safety or for pig health, are needed. Furthermore, it is known that different serotypes of *Salmonella* can have different dynamic of infection in the swine and hence could affect the metabolome differently [48]. Further experiments are required to know if the results obtained here are specific to the monophasic variant of *S. typhimurium* or can be generalized to other *Salmonella* serotypes. Finally, in this study, the molecules associated with metabolic signals were not identified. Indeed, although hypothetical identification using the signal mass alone is possible, tandem mass spectrometry is required to obtain identification with a high degree of confidence. Therefore, this study did not identify the metabolic pathway most affected by infection. However, even with these limitations, we have confidently demonstrated that infection, here with mST, can cause significant changes in the global animal’s serum metabolome, even in the absence of symptoms. 

## 6. Conclusions

In conclusion, we have shown for the first time the possibility to discriminate, in an experimental trial, animals infected by the monophasic variant of *Salmonella* Typhimurium both at the beginning of the infection and, although with less efficiency, later when no symptoms could be observed, on the basis of their overall serum metabolome using LC–HRMS. We also showed that although differences could be measured between infected and noninfected animals throughout the experiment, these differences evolved between the first and last day of the infection, leading to a possible significant difference between high- and low-shedding animals 21 days postinfection. Finally, we showed that there was a statistical association between infection-induced changes in certain bacterial populations present in the fecal microbiota and multiple serum metabolic signals. Therefore, the measurement of the serum metabolome changes seems to be a promising tool for the control of this zoonotic foodborne pathogen, *Salmonella*, in pigs. 

## Figures and Tables

**Table 1:** Performance values of PLS-DA models discriminating animals of different shedding groups by their serum metabolome composition at 1 and 21 DPI and for the positive and negative ionization mode.

Ionization Mode	Animal Shedding Status Comparison	DPI	R2Y	Q2	pR2Y	pQ2
Positive	Infected/Noninfected	1	0.997	0.955	0.002	0.002
		21	0.966	0.644	**0.098**	0.004
	High shedders/Low shedders/Noninfected	1	0.989	0.755	0.002	0.002
		21	0.872	**0.419**	0.01	0.002
	High shedders/Noninfected	1	0.99	0.891	0.004	0.002
		21	0.996	0.864	0.002	0.002
	Low shedders/Noninfected	1	0.999	0.955	0.004	0.002
		21	1	0.719	0.002	**0.062**
	High shedders/Low Shedders	1	0.99	0.696	**0.116**	0.02
		21	0.997	0.708	**0.238**	0.008
Negative	Infected/Noninfected	1	0.996	0.925	0.014	0.002
		21	0.989	0.754	0.018	0.004
	High shedders/Low shedders/Noninfected	1	0.98	0.757	0.002	0.002
		21	0.987	0.739	0.002	0.002
	High shedders/Noninfected	1	0.992	0.916	0.012	0.002
		21	0.997	0.891	0.014	0.002
	Low shedders/Noninfected	1	0.994	0.895	0.02	0.002
		21	0.998	0.724	0.016	0.044
	High shedders/Low Shedders	1	0.995	0.823	**0.084**	0.002
		21	0.995	0.708	0.024	0.002

Values in bold are under the acceptable quality score value (R2Y and Q2 < 0.5) or over the significant value threshold for the permutation test (*p* > 0.05).

**Table 2:** Performance values of PLS-DA models discriminating animals at 1 and 21 DPI by their serum metabolome composition for each shedding level group and for the negative and positive ionization mode.

Ionization Mode	Animal Shedding Status	R2Y	Q2	pR2Y	pQ2
Positive	Noninfected	0.996	0.535	**0.266**	**0.12**
	High shedders	0.997	0.939	0.002	0.002
	Low shedders	0.99	0.92	0.002	0.002
Negative	Noninfected	0.989	0.562	**0.162**	**0.062**
	High shedders	0.988	0.918	0.002	0.002
	Low shedders	0.993	0.931	0.002	0.002

Values in bold are under the acceptable quality score value (R2Y and Q2 < 0.5) or over the significant value threshold for the permutation test (*p* > 0.05).

**Table 3:** Performance values of PLS-DA models discriminating the animals that have become seropositive or not 21 days after infection by their serum metabolome composition.

Ionisation Mode	Animal Shedding Status	DPI	R2Y	Q2	pRY2	pQ2
Positive	All shedders	1	0.993	0.802	0.022	0.002
		21	0.996	0.633	**0.43**	0.026
	High shedders	1	0.998	0.728	**0.15**	**0.07**
		21	0.995	**0.489**	**0.272**	**0.152**
	Low shedders	1	0.965	**0.245**	**0.614**	**0.682**
		21	0.993	0.527	**0.648**	**0.28**
Negative	All shedders	1	0.897	**0.463**	**0.094**	0.026
		21	0.944	**0.371**	**0.556**	**0.088**
	High shedders	1	0.994	0.741	**0.202**	0.036
		21	0.989	0.518	**0.132**	**0.176**
	Low shedders	1	0.921	**0.311**	**0.492**	**0.548**
		21	0.995	**0.433**	**0.996**	**0.596**

Values in bold are under the acceptable quality score value (R2Y and Q2 < 0.5) or over the significant value threshold for the permutation test (*p* > 0.05).

**Table 4:** Alpha diversity comparisons of the fecal microbiota of pigs on the basis of their *Salmonella* shedding at 1- and 21-DPI.

	Days Postinfection	All Positive	High Shedder	Low Shedder	Negative
Observed OTUs	1 DPI	409 ^a^	433	381 ^b^	459 ^ab^
	21 DPI	620 ^a^	606 ^b^	635	665 ^ab^
Inverted Simpson’s	1 DPI	12.23	13.58	10.64	12.07
	21 DPI	31.974	32.91	30.95	28.34
Shannon evenness	1 DPI	0.635	0.645	0.623	0.6414
	21 DPI	0.732	0.728	0.736	0.737

Values with the same superscripts (a, b in a row are significantly different (*p* < 0.05)).

**Table 5:** Beta diversity comparison of the fecal microbiota of swine according to *Salmonella* infection status and shedding levels at 1 and 21 DPI.

Animal Infection Status Comparison	DPI	PERMANOVA (*p*-Value)
Infected/Noninfected	1	**0.037**
	21	**0.018**
High shedders/Low shedders/noninfected	1	0.119
	21	0.060
High shedders/Noninfected	1	0.071
	21	**0.048**
Low shedders/Noninfected	1	0.071
	21	**0.048**
High shedders/Low shedders	1	0.785
	21	0.539

Values in bold indicate significant PERMANOVA comparison (*p* < 0.05).

**Table 6:** Alpha diversity comparisons of the fecal microbiota of pigs at 1 DPI and 21 DPI for all the *Salmonella* shedding statuses.

	Shedding Level	1 DPI	21 DPI	*p*-Value
Observed OTUs	High shedders	433	606	**3 × 10^−5^**
	Low shedders	381	635	**3 × 10^−6^**
	Noninfected	459	665	**0.008**
Inverted Simpson’s	High shedders	13.58	32.91	**0.001**
	Low shedders	10.64	30.95	**6 × 10^−6^**
	Noninfected	12.07	28.34	**0.008**
Shannon evenness	High shedders	0.645	0.728	**6 × 10^−4^**
	Low shedders	0.623	0.736	**6 × 10^−6^**
	Noninfected	0.641	0.737	**0.008**

Values in bold indicate a significant Mann–Whitney comparison (*p* < 0.05).

**Table 7:** Beta diversity comparison of the fecal microbiota of pigs at 1 and 21 DPI according to shedding levels.

Animal Infection Status	PERMANOVA (*p*-Value)
Noninfected	**0.007**
High shedders	**0.000**
Low shedders	**0.000**

Values in bold indicate significant PERMANOVA comparison (*p* < 0.05).

**Table 8:** Alpha diversity comparisons of the fecal microbiota of pigs on the basis of their *Salmonella* seropositivity status at 21 DPI.

		Days Postinfection	Seronegative	Seropositive
Observed OTUs	Infected pigs	1 DPI	417	403
		21 DPI	630	613
	High shedder	1 DPI	417	446
		21 DPI	618	599
	Low shedder	1 DPI	416	352
		21 DPI	639	632
Inverted Simpson’s	Infected pigs	1 DPI	10.52	13.68
		21 DPI	32.68	31.52
	High shedder	1 DPI	9.6	16.98
		21 DPI	32.49	33.13
	Low shedder	1 DPI	11.63	9.82
		21 DPI	32.83	29.39
Shannon evenness	Infected pigs	1 DPI	0.625	0.643
		21 DPI	0.737	0.728
	High shedder	1 DPI	0.612	0.673
		21 DPI	0.730	0.727
	Low shedder	1 DPI	0.639	0.609
		21 DPI	0.743	0.730

**Table 9:** Beta diversity comparison of the fecal microbiota of seropositive and seronegative pigs at 21 DPI according to shedding levels.

Animal Shedding Status	PERMANOVA (*p*-Value)	
	1 DPI	21 DPI
All shedders	0.619	0.198
High shedders	0.231	0.927
Low shedders	0.783	0.123

## Data Availability

Raw sequencing dataset is available on the Dryad repository https://doi.org/10.5061/dryad.wwpzgmsm3.

## Supplementary Materials

The following supporting information can be downloaded at: https://www.mdpi.com/article/10.3390/microorganisms11102565/s1, Supplementary Figure S1: PLS-DA plots for the classification of infected and noninfected animals using the serum metabolome composition in positive acquisition mode (A) 1 DPI and (B) 21 DPI and negative acquisition mode (C) 1 DPI and (D) 21 DPI; Supplementary Figure S2: Graphs of the permutation tests’ results for PLS-DA models classifying infected and noninfected animals using the serum metabolome composition in positive acquisition mode (A) 1 DPI and (B) 21 DPI and negative acquisition mode (C) 1 DPI and (D) 21 DPI; Supplementary Figure S3: PLS-DA plots for the classification, using the serum metabolome composition, of low shedders, high shedders and noninfected animals in negative acquisition mode (A) 1 DPI and (B) 21 DPI; Supplementary Figure S4: Graphs of the permutation tests’ results for PLS-DA models classifying low shedders, high shedders and noninfected animals in negative acquisition mode (A) 1 DPI and (B) 21 DPI; Supplementary Figure S5: PLS-DA plots for the pairwise classification using the serum metabolome composition of low shedders, high shedders and noninfected animals in negative acquisition mode (A, C, E) 1 DPI and (B, D, F) 21 DPI; Supplementary Figure S6: Graphs of the permutation tests’ results for PLS-DA models for the pairwise comparisons of the serum metabolome composition of low shedders, high shedders and noninfected animals in negative acquisition mode (A, C, E) 1 DPI and (B, D, F) 21 DPI; Supplementary Figure S7: PLS-DA plots for classification using the serum metabolome composition of animals at 1 and 21 DPI in negative acquisition mode for high shedder (A), low shedder (B) and noninfected (C); Supplementary Figure S8: Graphs of the permutation tests’ results for PLS-DA models for classification using the serum metabolome composition of animals at 1 and 21 DPI in negative acquisition mode for high shedder (A), low shedder (B) and noninfected (C); Supplementary Figure S9: PCA graphs comparing the serum metabolome at 1 DPI (all positive (A), high shedders (C), low shedders (E)) and 21 DPI (all positive (B), high shedders (D), low shedders (F)) of animals that are seropositive or not at 21 DPI in negative acquisition mode; Supplementary Figure S10: PLS-DA plots for classification using the serum metabolome at 1 DPI (all positive (A), high shedders (C), low shedders (E)) and 21 DPI (all positive (B), high shedders (D), low shedders (F)) of animals that are seropositive or not at 21 DPI in negative acquisition mode; Supplementary Figure S11: Graphs of the permutation tests’ results for PLS-DA models for classification using the serum metabolome at 1 DPI (all positive (A), high shedders (C), low shedders (E)) and 21 DPI (all positive (B), high shedders (D), low shedders (F)) of animals that are seropositive or not at 21 DPI in negative acquisition mode;Supplementary Figure S12: Network representation of the sPLS regression-measured association of the fecal microbiota and serum metabolomic signals variations between infected and noninfected animals in negative acquisition mode (A) 1 DPI and (C) 21 DPI and positive acquisition mode (B) 1 DPI and (D) 21 DPI; Supplementary Figure S13: Network representation of the sPLS regression-measured association of the fecal microbiota and serum metabolomic signals variations between high shedders and noninfected animals in negative (A) and positive (B) acquisition mode and between low shedders and noninfected animals in negative (C) and positive (D) acquisition mode; Supplementary Figure S14: Network representation of the sPLS regression-measured association of the fecal microbiota and serum metabolomic signals variations between 1 and 21 DPI for high-shedding animals in negative (A) and positive (B) acquisition mode and low shedders and noninfected animals in negative (C) and positive (D) acquisition mode; Supplementary Table S1 sPLS-measured associations between OTUs and metabolic signals which vary when comparing *Salmonella*-infected and noninfected animals and the intensity of these associations at 1 DPI in positive acquisition mode; Supplementary Table S2: sPLS-measured associations between OTUs and metabolic signals which vary when comparing *Salmonella*-infected and noninfected animals and the intensity of these associations at 21 DPI in positive acquisition mode; Supplementary Table S3: sPLS-measured associations between OTUs and metabolic signals which vary when comparing *Salmonella*-infected and noninfected animals and the intensity of these associations at 1 DPI in negative acquisition mode; Supplementary Table S4: sPLS-measured associations between OTUs and metabolic signals which vary when comparing *Salmonella*-infected and noninfected animals and the intensity of these associations at 21 DPI in negative acquisition mode; Supplementary Table S5: sPLS-measured associations between OTUs and metabolic signals which vary when comparing high *Salmonella* shedders with noninfected animals at 21 DPI in positive acquisition mode and the intensity of these associations; Supplementary Table S6: sPLS-measured associations between OTUs and metabolic signals which vary when comparing high *Salmonella* shedders with noninfected animals at 21 DPI in negative acquisition mode and the intensity of these associations; Supplementary Table S7: sPLS-measured associations between OTUs and metabolic signals which vary when comparing low *Salmonella* shedders with noninfected animals at 21 DPI in positive acquisition mode and the intensity of these associations; Supplementary Table S8: sPLS-measured associations between OTUs and metabolic signals which vary when comparing low *Salmonella* shedders with noninfected animals at 21 DPI in negative acquisition mode and the intensity of these associations; Supplementary Table S9: sPLS-measured associations between OTUs and metabolic signals which vary when comparing high-shedding *Salmonella*-infected animals at 1 and 21 DPI in negative acquisition mode and the intensity of these associations; Supplementary Table S10: sPLS-measured associations between OTUs and metabolic signals which vary when comparing low-shedding *Salmonella*-infected animals at 1 and 21 DPI in negative acquisition mode and the intensity of these associations; Supplementary Table S11: sPLS-measured associations between OTUs and metabolic signals which vary when comparing high-shedding *Salmonella*-infected animals at 1 and 21 DPI in positive acquisition mode and the intensity of these associations; Supplementary Table S12: sPLS-measured associations between OTUs and metabolic signals which vary when comparing low-shedding *Salmonella* infected animals at 1 and 21 DPI in positive acquisition mode and the intensity of these associations.
